# Cerium Dioxide Particles to Tune Radiopacity of Dental Adhesives: Microstructural and Physico-Chemical Evaluation

**DOI:** 10.3390/jfb11010007

**Published:** 2020-02-11

**Authors:** Isadora Martini Garcia, Vicente Castelo Branco Leitune, Antonio Shigueaki Takimi, Carlos Pérez Bergmann, Susana Maria Werner Samuel, Mary Anne Melo, Fabrício Mezzomo Collares

**Affiliations:** 1 Dental Materials Laboratory, School of Dentistry, Federal University of Rio Grande do Sul, Rua Ramiro Barcelos 2492, Rio Branco, Porto Alegre, RS 90035-003, Brazil; isadora.garcia@ufrgs.br (I.M.G.); vicente.leitune@ufrgs.br (V.C.B.L.); susana.samuel@ufrgs.br (S.M.W.S.); 2 Laboratory for Electrochemical Processes and Corrosion, Engineering School, Federal University of Rio Grande do Sul, Bento Gonçalves, 9500, Prédio 43427, Sala 216, Porto Alegre, RS 91501-970, Brazil; antonio.takimi@gmail.com; 3 Laboratory of Ceramic Materials, Federal University of Rio Grande do Sul, Avenida Osvaldo Aranha 99, Porto Alegre, RS 90035-003, Brazil; bergmann@ufrgs.br; 4 Division of Operative Dentistry, Department of General Dentistry, University of Maryland School of Dentistry, Baltimore, MD 21201, USA; 5 Ph.D. Program in Biomedical Sciences, University of Maryland School of Dentistry, Baltimore, MD 21201, USA

**Keywords:** dental materials, dentistry, adhesives, light-curing of dental adhesives, composite resins, methylmethacrylate, oxides, cerium, polymers, dentine bonding agents

## Abstract

The insufficient radiopacity of dental adhesives applied under composite restorations makes the radiographic diagnosis of recurrent caries challenging. Consequently, the misdiagnosis may lead to unnecessary replacement of restorations. The aims of this study were to formulate experimental dental adhesives containing cerium dioxide (CeO_2_) and investigate the effects of different loadings of CeO_2_ on their radiopacity and degree of conversion for the first time. CeO_2_ was characterized by X-ray diffraction analysis, Fourier transforms infrared spectroscopy, and laser diffraction for particle size analysis. Experimental dental adhesives were formulated with CeO_2_ as the inorganic filler with loadings ranging from 0.36 to 5.76 vol.%. The unfilled adhesive was used as a control. The studied adhesives were evaluated for dispersion of CeO_2_ in the polymerized samples_,_ degree of conversion, and radiopacity. CeO_2_ presented a monoclinic crystalline phase, peaks related to Ce-O bonding, and an average particle size of around 16 µm. CeO_2_ was dispersed in the adhesive, and the addition of these particles increased the adhesives’ radiopacity (*p* < 0.05). There was a significant decrease in the degree of conversion with CeO_2_ loadings higher than 1.44 vol.%. However, all materials showed a similar degree of conversion in comparison to commercially available adhesives. CeO_2_ particles were investigated for the first time as a promising compound to improve the radiopacity of the dental adhesives.

## 1. Introduction

Currently, resin composites and dental adhesive systems are used in restorative dentistry as primary direct restorative materials [1]. The failure of resin composites is mainly due to recurrent caries and fractures [2]. The diagnosis of recurrent caries underneath resin composite is a challenge for dentists [3]. Recurrent carious lesions may not always be seen during a clinical examination at the interface between the resin composite, and it requires radiographic evaluation for diagnosis [4,5].

Further, the application of adhesive systems can be seen radiographically as a radiolucent area, which mimics the radiographic appearance of carious lesions [6]. The radiolucency of dental adhesives can contribute to the misinterpretation of radiographic images [4,7,8]. Based on it, dentists may intervene surgically in existing composite restorations, replacing the resin [9].

A previous report has assessed and demonstrated the lack of radiopacity on many restorative dental materials [10]. In their results, all assessed materials were radiolucent and required alterations to their composition to facilitate their detection using radiographic images. The radiopacity of dental adhesives depends on their filler content, and it can be enhanced by incorporating elements with a high atomic weight as inorganic fillers [11]. 

Cerium dioxide (CeO_2_), a rare-earth oxide found in the lanthanide series of the periodic table, has been increasingly used as a nanotherapeutic material [12]. The numerous commercial applications for CeO_2_ also called ceria, include glass and glass polishing, phosphors, ceramics, catalysts, and metallurgy [13]. Cerium is found in various minerals, and its primary deposits are located in the United States (Florida and Idaho) and Brazil [14]. Interesting biological properties have been observed for both nanometric and micrometric CeO_2_ [15]. Thus, as further advancement in CeO_2_′s applications, this compound has gained substantial interest in several innovative applications, mainly due to its redox property and catalytic activity [15,16]. In the dental field, CeO_2_ was primarily used for dental ceramics since this compound stimulates the natural fluorescence found in human dental enamel [11]. The high atomic number 58 of cerium suggests that it can promote considerable attenuation of a dental X-ray beam [17,18]. The incorporation of CeO_2_ in adhesives can be a valuable strategy to promote radiopacity and improve the detection of dental adhesives underneath resin composites. Therefore, the aims of this study were to formulate experimental dental adhesives containing CeO_2_ and investigate the effects of different loadings of CeO_2_ on their radiopacity and degree of conversion for the first time.

## 2. Results

**Figure 1:**
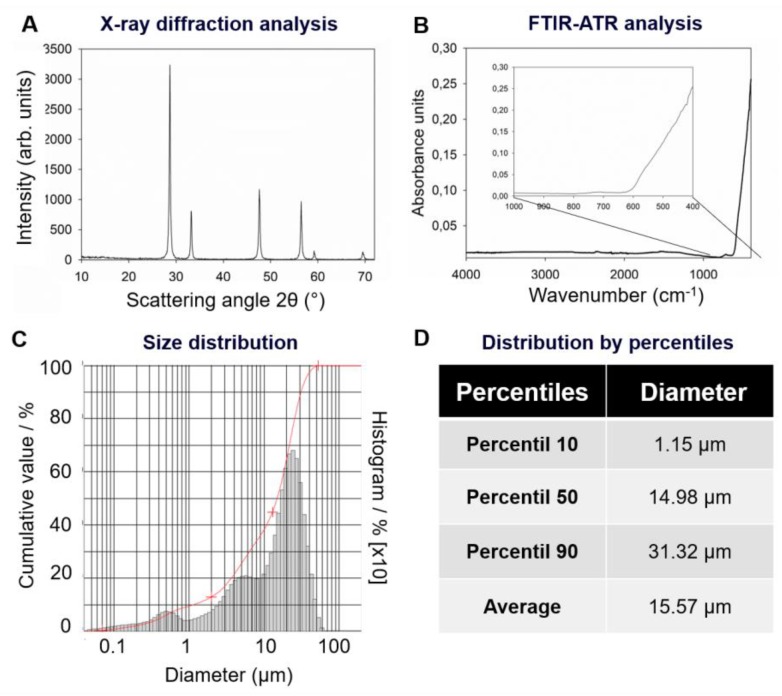
CeO_2_ particles’ characterization: (**A**) X-ray diffraction analysis shows the pattern of the monoclinic phase of crystallinity for CeO_2_; (**B**) FTIR analysis displays the peaks related to Ce-O bonds at 400 cm^−1^; (**C)** and (**D**) show the non-normal distribution size of CeO_2_ and the values of size distribution.

Figure 1 shows the results of CeO_2_ particles’ characterization. In Figure 1A, the X-ray diffraction analysis of the powder shows the crystallinity pattern of a monoclinic phase of CeO_2_ (powder diffraction file, ICDD-PDF 37-1468). In Figure 1B, the chemical composition analyzed by Fourier Transform Infrared Spectroscopy (FTIR) displays the peaks related to Ce–O bonds at 400 cm^−1^. Figure 1C,D presents the results of size distribution analysis. The histogram shows a non-normal distribution of size (Figure 1C). The 10th percentile showed a particle diameter of 1.15 µm. The median (50th percentile) was 14.98 µm, the 90th percentile was 31.32 µm, and the mean particle size was around 16 µm (Figure 1D).

**Figure 2:**
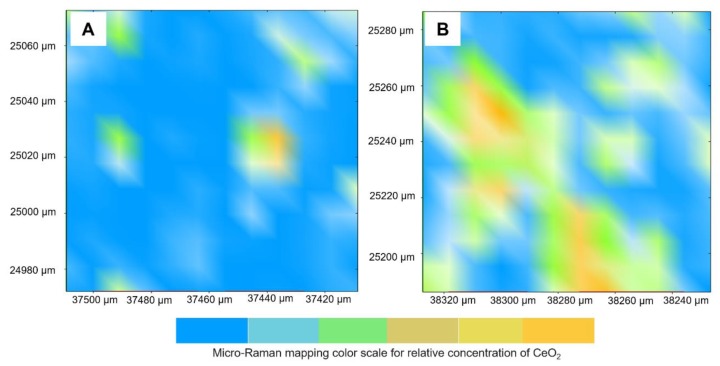
Micro-Raman analysis of the dental adhesive surfaces containing 0.36 (**A**) and 0.76 (**B**) vol.% of CeO_2_. Both images display the intensity of the CeO_2_ peak at 464 cm^−1^. The higher the load of CeO_2_ in the dental adhesive, the more areas in yellow are observed.

Figure 2 presents the qualitative assessment via micro-Raman of the materials’ surfaces containing 0.36 (A) and 0.76 (B) vol.% of CeO_2_. The image consists of a 2-D array of measured spectra, which means that the distribution of the chemical composition can be investigated. After mapping the polymer’s surfaces, the integration of the corresponding CeO_2_ peak (464 cm^−1^) was used for analysis and graphs generation. In the graphs, from blue to yellow, more CeO_2_ is identified. It was observed that the higher the load of CeO_2_ in the resin, the larger the area under the curve (peak) in micro-Raman spectra for this oxide, generating more yellow regions in the graph. In these analyses, more areas in yellow were presented in the adhesive with 0.76 vol.% of CeO_2_ in comparison to the group with 0.36 vol.%.

**Figure 3:**
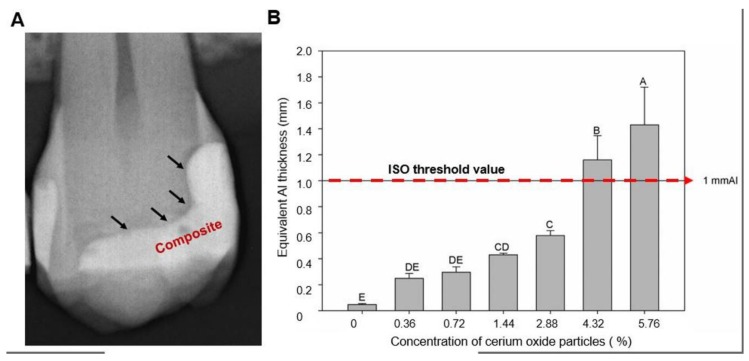
The dental radiograph displays the layer of dental adhesive applied under the composite resin (**A**) The arrows guide the visualization of the radiolucent adhesive layer. (**B**) The radiopacity of the experimental dental adhesives according the to the increasing concentration of cerium oxide. Different letters indicate statistical differences among groups (*p* < 0.05).

Figure 3 indicates the results of the radiopacity of each experimental adhesive resin containing a different percentage of volume fraction of CeO_2_. In Figure 3A, an illustrative radiograph displays the location of the dental adhesive layer underneath the composite restorative material. The arrows indicate the radiolucent areas corresponding to the adhesive layer. The difference of radiopacity between the composite resin and the adhesive layer can be clearly observed. In Figure 2B, the mean and standard deviation of radiopacity is expressed in mm of aluminum. The control group, which is an unfilled adhesive, showed the lower mean value of radiopacity, without statistical difference for the groups with 0.36 vol.% and 0.72 vol.% of CeO_2_. From the addition of 1.44 vol.% of CeO_2_, there was increased radiopacity of the adhesive in comparison to the control group (*p* < 0.05). The group with 5.76 vol.% of CeO_2_ showed the highest value of radiopacity among all groups (*p* < 0.05). The incorporation of 4.32 vol.% and 5.76 vol.% presented values of more than 1 mm of aluminum.

**Figure 4:**
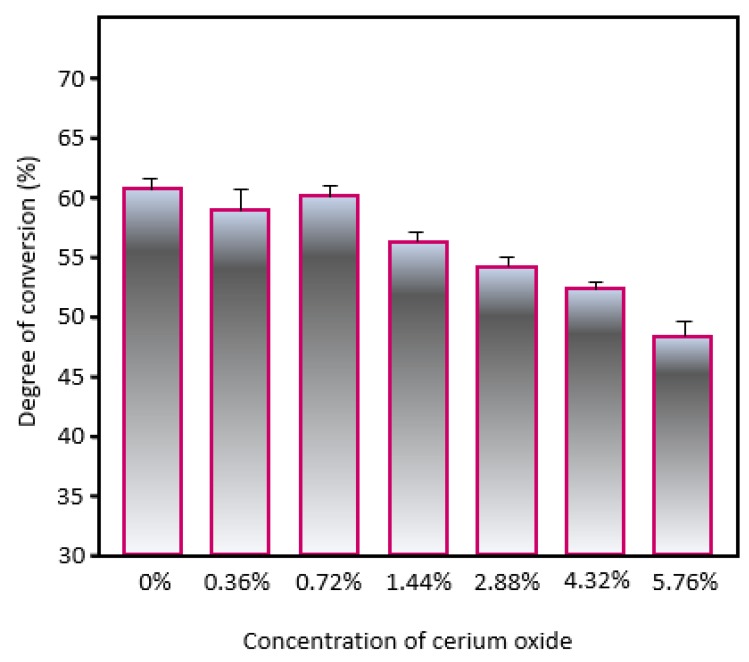
Degree of conversion of the experimental dental adhesives containing CeO_2_. Different letters indicate statistical differences among groups (*p* < 0.05).

Figure 4 shows the results of the degree of conversion analysis of the experimental dental adhesives. The uncured adhesive samples were directly dispensed on the attenuated total reflection (ATR) device of FTIR to analyze the conversion of carbon-carbon double bonds in the aliphatic chain. The values ranged from 61.52 (±0.33) % for the control group to 47.90 (±1.64) % for 5.76 vol.% of CeO_2_. From the incorporation of 2.88 vol.% of CeO_2_, the degree of conversion reduced in comparison to the control group (*p* < 0.05). The lowest value of the degree of conversion was found for the highest load of CeO_2_ incorporated in the adhesive (*p* < 0.05).

## 3. Discussion

In this in vitro study, CeO_2_ particles were explored as potential radiopacifier for dental adhesives since high atomic weight and density can provide a suitable level of radiopacity. CeO_2_ was first chemically characterized by XRD, Raman, and FTIR to be incorporated for the first time in a dental adhesive resin. In the current investigation, CeO_2_ successfully increased the radiopacity of the adhesive, maintaining a suitable degree of monomer conversion.

CeO_2_ particles used in this study presented a monoclinic crystalline phase with particular chemical groups and the average particle size of around 16 µm. This oxide was incorporated in an experimental adhesive resin formulated as previously reported, with conventional dental methacrylate monomers [19,20]. The presence of a radiopacifying agent is essential to enable the identification of restorative materials and dissimilarity from pathological processes in the adjacent areas [21]. Recurrent caries and marginal gaps are very often the reasons for the replacement of composite restorations [22]. The misdiagnosis and treatment decision for unnecessary replacement of restorations lead to additional loss of sound tooth tissue, with an increased cost and discomfort for the patient [23]. Therefore, the restorative materials should have optimal radiopacity for accurate radiographic differentiation for existing restorations and recurrent dental caries, supporting clinical follow-ups. Substantial changes in radiopacity are compulsory by the International Organization for Standardization (ISO) for dental restorative materials [24]. The compliance with the ISO 4049 requires a minimum radiopacity of restorative materials higher than that of dentin and greater or equivalent thickness of aluminum (with ≥98% purity) [24]. In the present study, the increased load of CeO_2_, by the percentage of volume, was added to an experimental dental adhesive, providing a material with proper radiopacity. Our results have shown that CeO_2_ at 4.32 vol.% had radiopacity higher than 1 mm of Al.

Besides the increase of radiopacity, the addition of inorganic fillers in monomeric blends may improve polymers properties, such as the elastic modulus, tensile strength, fracture toughness, Knoop hardness, and stability against solvents [25]. However, the higher filler addition can also decrease the degree of monomer’s conversion [19]. The degree of conversion is a valuable chemical property for polymers, and it may be associated with the stability of the restoration over time [20]. Therefore, a reliable polymerization is desired for adhesive resins to reduce hydrolytic degradation in the clinical setting [26,27]. Here, we observed that the incorporation of CeO_2_ at a load that reaches the high radiopacity level reduced the degree of conversion of the dental polymer. Previous studies have reported similar outcomes in the investigations of radiopacifying agents containing different kinds of heavy metals [28]. Marins et al. [29] evaluated the addition of niobium pentoxide (Nb_2_O_5_) nanoparticles to the dental adhesive as radiopacifiers and observed that the degree of conversion decreased with the addition of particles at percentage mass fraction equal or greater than 10%. In other studies, Nb_2_O_5_ and Ta_2_O_5_ showed a decrease in the degree of conversion up to 5 wt.% [19,20]. Our results are also in agreement with those of Amirouche-Korichi et al. [28], where progressive decreases of the degree of conversion were linearly related to the filler contents.

The outcome observed for the degree of conversion may be attributed to the high refractive index of CeO_2_ (approx. ղ = 2.2 to 2.8) [30], which may have decreased the accessibility of light energy inside the polymer. The limited mobility of monomer chains by the incorporation of the opaque fillers has also been considered as a contributing factor for the decrease in the monomer’s conversion [31]. Another consideration for observed decay in the degree of conversion with regard to the dental adhesive with loadings higher than 2.88 vol.% relies on particle size. The fillers used in our study are micro fillers with an average size of around 16 µm. Previous reports support the effect of particle size in microns and highlight that the volume occupied by the particles may compromise the polymerization rates of the material [32,33,34]. Further evaluation of the polymerization behavior of nanosized CeO_2_ could be interesting to address this subject. Moreover, it would be noteworthy to evaluate the effect of micro-sized CeO_2_ in the adhesive with thinner samples or in situ in dentin.

Moreover, we suggest further biological studies on dental adhesives containing CeO_2_ in micro and nanoscale, since this oxide has emerged as a noteworthy agent for bioactive (such as scaffolds and bioglasses) and antimicrobial materials [35,36,37]. In this context, a dental adhesive with such properties could be a promising strategy to assist in decreasing the incidence of recurrent caries and improving the remineralization process after selective removal of carious dentin. In this study, despite the decreased degree of conversion observed by increasing CeO_2_ addition, all groups showed values around 50%, which is in accordance with commercial dental adhesives [38]. Therefore, CeO_2_ may be a promising alternative filler for biopolymers.

## 4. Materials and Methods

### 4.1. X-ray Diffraction Analysis of CeO_2_

CeO_2_ particles purchased were analyzed via X-ray diffraction to detect the crystalline phases of the powder. diffractometer (PW 1730/1 model, Philips, Santa Clara, CA, USA) was operated at 40 kV and 40 mA with CuKa radiation. The scanning rate used was 0.058/min, with 2 s of time-steps at 0.02° each, from 5° to 60° [18].

### 4.2. FTIR Analysis of CeO_2_

Fourier Transform Infrared Spectroscopy (FTIR) was used to chemically characterize the powder of CeO_2_. The analysis was performed using the spectrophotometer Vertex 70 (Bruker Optics, Ettlingen, Germany) with an attenuated total reflectance device (ATR). CeO_2_ powder was placed on the ATR, and the analysis performed using 20 scans, 4 cm^−1^ of resolution in 5000 to 400 cm^−1,^ and Opus 6.5 software (Bruker Optics, Ettlingen, Germany).

### 4.3. Particle Size Distribution of CeO_2_

CeO_2_ particles were dispersed in water with sonication for 60 s. Then, particle size was analyzed via laser diffraction particle size analyzer (CILAS 1180, Cilas, Orleans, France) according to a previous study [18].

### 4.4. Preparation of Dental Adhesives

The adhesive resins were formulated by mixing 50 wt.% bisphenol A glycol dimethacrylate (BisGMA), 25 wt.% triethylene glycol dimethacrylate (TEGDMA), and 25 wt.% 2-hydroxyethyl methacrylate (HEMA). As a photoinitiator system, camphorquinone and ethyl 4-dimethylaminobenzoate were added at 1 mol%, each one in the base resin. As polymerization inhibition, 0.01 wt.% of butylated hydroxytoluene was incorporated. CeO_2_ was added at 0.36, 0.72, 1.44, 2.88, 4.32, and 5.76 vol.%. The resins adhesives were hand-mixed for 5 min, sonicated during 180 s, and hand-mixed again for 5 min. One group without CeO_2_ was used as control. 

### 4.5. Qualitative Analysis of CeO_2_ into Dental Adhesives

To identify the presence of CeO_2_ in dental adhesives, two groups containing this filler were evaluated via micro-Raman Spectroscopy (Senterra, Bruker Optics, Ettlingen, Germany). One sample from the group with 0.36 vol.% and another from the group with 5.76 vol.% were prepared using a polyvinylsiloxane mold. The samples were photoactivated for 20 s on each side using a light-emitting diode dental curing unit (Radii Cal, SDI, Melbourne, Australia) with 1200 mW/cm^2^. An area of 110 µm × 110 µm of the surfaces was analyzed via Opus 7.5 software (Opus 7.5, Bruker Optics, Ettlingen, Germany). The analyses were performed with a wavelength of 785 nm, with 5 s and two co-additions. The peak correspondent to CeO_2_ (464 cm^−1^) was used for integration.

### 4.6. Radiopacity Evaluation

The adhesive resins were evaluated for their radiopacity, according to the International Organization of Standardization (ISO) 4049/2009 guidelines [24]. Five samples per group (n = 5) with disc-shaped were prepared with 10.0 mm (±0.5 mm) in diameter and 1.0 mm (±0.1 mm) in thickness with photoactivation for 20 s on each side. The images were made using a phosphor plate for the digital system (VistaScan; Dürr Dental GmbH & Co. KG, Bietigheim-Bissingen, Germany) at 70 kV, 8 mA, and 0.4 s of exposure time. A focus-film distance of 400 mm was used in the assays. One specimen per group was positioned on the film for each X-ray exposition. An aluminum step-wedge (99.12 wt.% of aluminum, thickness from 0.5 mm to 5.0 mm, in increments of 0.5 mm) was exposed with the samples for each image specimens in all images. The images were saved in TIFF, analyzed using Photoshop software (Adobe Systems Incorporated, San Jose, CA, USA), and the mean and standard deviation of the grey levels (pixel density) were measured.

### 4.7. Degree of Conversion

To analyze the degree of conversion of the adhesives, FTIR-ATR was used according to a previous study [39]. Three drops per group (n = 3, 3 µL each one) were directly dispensed on ATR diamond crystal. An adjustable holder stand was used to fix the light-curing unit and to standardize the distance between its tip and the top of the samples. Two spectra were acquired per group from 4000 to 400 cm^−1^ with a resolution of 4 cm^−1^. The first spectrum was obtained before the photoactivation. Then, the samples were photoactivated for 20 s and analyzed again. To calculate the monomer conversion, the peak related to the carbon-carbon (C=C) double bond in the aromatic ring of BisGMA (1610 cm^−1^) was used as an internal standard. The peak related to C=C in the aliphatic chains (1640 cm^−1^) was used along with the C=C at 1610 cm^−1^ according to the following equation:

$$\mathrm{Degree}\;\mathrm{of}\;\mathrm{conversion}\;\left(\%\right)=100\times \left(\frac{I\_\;1640-\mathrm{cured}/I\_\;1610-\mathrm{cured}}{I\_\;1640-\mathrm{uncured}/I\_1610-\mathrm{uncured}}\right)$$

### 4.8. Statistical Analysis

CeO_2_ characterization was descriptively analyzed, as well as the identification of CeO_2_ in the polymerized adhesives. Shapiro-Wilk test was used to evaluate the data normality. The data of radiopacity was evaluated via ANOVA on ranks and Dunn’s test. The data of the degree of conversion was analyzed via one-way ANOVA and Tukey’s post-hoc test. Both analyses were performed at 0.05 level of significance (*p* < 0.05).

## 5. Conclusions

CeO_2_ was investigated for the first time as a promising filler to improve the radiopacity of dental adhesives. The particles were chemically analyzed and then used in experimental adhesives formulation. Through of incorporation of CeO_2_ particles at 1.44 vol.%, the dental adhesives were able to show increased radiopacity and a proper degree of conversion. The restorative materials here proposed could be a reliable strategy to assist clinicians diagnose recurrent caries.
